# Albuminuria detection using graphene oxide-mediated fluorescence quenching aptasensor

**DOI:** 10.1016/j.mex.2020.101114

**Published:** 2020-10-20

**Authors:** Wireeya Chawjiraphan, Chayachon Apiwat, Khoonsake Segkhoonthod, Kiatnida Treerattrakoon, Preedee Pinpradup, Nuankanya Sathirapongsasuti, Prapasiri Pongprayoon, Patraporn Luksirikul, Patcharee Isarankura-Na-Ayudhya, Deanpen Japrung

**Affiliations:** a National Nanotechnology Center (NANOTEC), National Science and Technology Development Agency (NSTDA), Thailand Science Park, Pathumthani, Thailand; b Section of Translational Medicine, Faculty of Medicine Ramathibodi Hospital, Mahidol University, Bangkok, Thailand; c Department of Chemistry, Faculty of Science, Kasetsart University, Bangkok, Thailand; d Center for Advanced Studies in Nanotechnology for Chemical, Food and Agricultural Industries, KU Institute for Advanced Studies, Kasetsart University, Bangkok, Thailand.; e Department of Medical Technology, Faculty of Allied Health Science, Thammasat University, Pathumthani, Thailand; f Department of Pure and Applied Chemistry, Technology and Innovation Centre, University of Strathclyde, Glasgow, United Kingdom

**Keywords:** Albuminuria, Human serum albumin, Chronic kidney disease, Aptasensor, Graphene oxide, Fluorescence quenching

## Abstract

A simple and sensitive graphene oxide-mediated fluorescence quenching aptasensor is developed to quantify albuminuria in urine samples. The developed aptasensor used the specific target binding property of aptamer and fluorescence quenching property of graphene oxide to determine the concentration of human serum albumin in urine. The limit of detection of the developed platform is 0.05 µg.mL^−1^ and the detection range is 0.1–600 µg.mL^−1^, which covers the albuminuria concentration range present in normal human urine and the urine of the patient with chronic kidney disease. This approach can be modified to measure albuminuria using a high-throughput quantification platform and portable point of care testing. In addition, the production cost for one reaction is cheaper than those for the standard automated method. Therefore, this aptasensor has significant potential for commercialization and public use.

• Our protocol is customized by using the fluorescence quenching property of graphene oxide and specific binding property of human serum albumin aptamer to detect human serum albumin in urine sample
• The limit of detection of our developed platform is 0.05 µg.mL^−1^
• The detection range of our aptasensor is 0.1–600 µg.mL^−1^

## Graphical abstract

**Figure:**
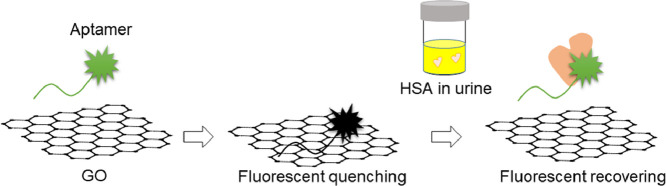

Specifications table

**Table:** 

Subject Area	Biochemistry, Genetics and Molecular Biology
More specific subject area	Nanotechnology for medical application
Method name	Albuminuria detection by graphene oxide-mediated fluorescence quenching aptasensor.
Name and reference of original method	(1) C. Apiwat, P. Luksirikul, P. Kankla, P. Pongprayoon, K. Treeratrakoon, K. Paoboonsukwong, S. Fucharoen, T. Dharakul, D. Japrung, Graphene based aptasensor for glycated albumin in diabetes mellitus diagnosis and monitoring, Biosens. Bioelectron. 82 (2016) 140-145. (2) W. Chawjiraphan, C. Apiwat, K. Segkhoonthod, K. Treerattrakoon, P. Pinpradup, N. Sathirapongsasuti, P. Pongprayoon, P. Luksirikul, P. Isarankura-Na-Ayudhya, D. Japrung, Sensitive detection of albuminuria by graphene oxide-mediated fluorescence quenching aptasensor. Spectrochimica Acta Part A: Molecular and Biomolecular Spectroscopy. Ms. Ref. No.: SAA-D-19-02819 (Spectrochim Acta A Mol Biomol Spectrosc. 2020 Apr 15;231:118128. doi: 10.1016/j.saa.2020.118128)
Resource availability	*If applicable, include links to resources necessary to reproduce the method (e.g. data, software, hardware, reagent)* • DNA aptamer can be ordered from https://www.idtdna.com/pages • Human serum albumin can be ordered from https://www.sigmaaldrich.com/singapore.html

## Method details

### Preparation of fluorescence-labeled aptamer and urine samples

The albumin binding aptamer used in this paper was the 87-nucleotide ssDNA, which was selected based on our previous work [1], and was an HPLC grade material synthesized by Integrated DNA Technologies, Singapore. The sequence of the DNA aptamer was 5’/Cy5/ATA CCA GCT TAT TCA ATT CCC CCG GCT TTG GTT TAG AGG TAG TTG CTC ATT ACT TGT ACG CTC CGG ATG AGA TAG TAA GTG CAA TCT/3’. The fluorescence-labeled aptamers were dissolved in sterile water to make a stock solution of 100 µM, which was aliquoted and kept at -20°C until future use. The purified human albumin (A9731) was purchased from Sigma-Aldrich (St. Louis, Missouri, USA). The reduced GO monolayer powder was prepared using Hummers method and dissolved in sterile water to obtain 5 mg.mL^−1^ solution as described in our previous study [2]. The phosphate buffered saline (PBS) consisted of 137 mmol.L^−1^ NaCl, 2.7 mmol.L^−1^ KCL, 10 mmol.L^−1^ Na_2_HPO_4_, and 1.8 mmol.L^−1^ KH_2_PO_4_ (pH 7.4), and was autoclaved at 121°C for 15 min and kept at room temperature until use.

One hundred and twenty urine samples were collected from the Faculty of Medicine, Ramathibodi Hospital, Mahidol University, Bangkok, Thailand, under the Research Network of NANOTEC (RNN) program with the MTA agreement (P1851893, ethical approval no. COA. MURA2019/796). The random spot urine samples were collected in sterile screw cap tubes, aliquoted, and stored at −80°C until these were used further.

### Optimization of aptamer concentration

As the aptamer used in this study was different from our previous report [1], the concentration of aptamer used in the system was first optimized. The 15 µL of 5 mg.mL^−1^ GO was mixed with 15 µL of the H8 aptamer of various concentrations (0–10 µM) and incubated at room temperature (25°C) for 5 min in the dark. Thereafter, 2 µL of 3 mg.mL^−1^ HSA was added into the mixed complex, the volume was adjusted to 200 µL, and incubated at room temperature for 30 min, then the fluorescence intensity was measured with excitation at 630 nm and emission at 670 nm using a Quantus portable fluorometer (Promega Corp., Madison, Wisconsin, USA). The optimal aptamer concentration was the concentration that completely quenched the fluorescence intensity of the system.

### Aptasensor performance for HSA measurement

The GO-mediated fluorescence quenching aptasensor was modified compared to our previous study [2]. In brief, the fluorescence-labeled aptamer was bound to GO and the fluorescence signal was quenched. When the target molecule was added to the system, the fluorescence-labeled aptamer detached the GO to bind to the target molecule and the fluorescence signal was recovered. The modified protocol was started by measuring fluorescence signals of purified HSA protein at various concentrations (0.001–0.6 mg.mL^−1^) in PBS buffer and plotting calibration curve. For standard curve analysis, 15 µL of 5 mg.mL^−1^ GO was mixed with 15 µL of 5 µM H8 aptamer and incubated at room temperature (25°C) for 5 min in the dark. Thereafter, 2 µL of purified HSA protein was added into the mixed complex, the volume was adjusted to 200 µL by adding PBS buffer, and the fluorescence intensity was measured with excitation at 630 nm and emission at 670 nm. Then the subtracted fluorescence intensity (ΔF) was obtained from Eq. 1 and plotted against the concentrations of HSA, linear regression equation was applied, and the LOD (3.3 × SD/Slope) was calculated.

(1) ΔF=Fob−Fmin

Whereas *F_ob_* is the fluorescence intensity at various HSA concentrations, *F_min_* is the fluorescence intensity of the aptamer-bound GO (GO–aptamer complex) in condition without HSA, which is the negative control.

### Evaluation of the aptasensor for albuminuria detection in urine samples

The HSA concentrations in urine samples were analyzed using the immunoturbidimetry method (Architect i2000SR, Abbott Laboratories), which was the standard method used in the hospital, and compared with the corresponding results of the aptasensor developed in this study. To obtain HSA concentration in urine samples, 15 µL of 5 mg.mL^−1^ GO was mixed with 15 µL of 5 µM H8 aptamer and incubated at room temperature (25°C) for 5 min in the dark. Thereafter, 2–100 µL of urine sample was added into the mixed complex, the volume was adjusted to 200 µL by adding PBS buffer, and the fluorescence was measured (excitation at 630 nm and emission at 670 nm). Then the subtracted fluorescence intensity (ΔF) was calculated and HSA concentration was determined using the linear equation from the calibration curve.

The Pearson correlation coefficients (*r*) and *p* values were determined using the SPSS Statistics version 20.0 software and Origin version 6.0 software. The correlation of two data sets was determined based on the *p* value. If the *p* values were < 0.01, these two data sets were considered to be statistically significantly correlated. Contrarily, if the *p* values were ≥ 0.01, these data sets were not considered to be correlated.

## Method validation results

### Optimization results

**Fig. 1:**
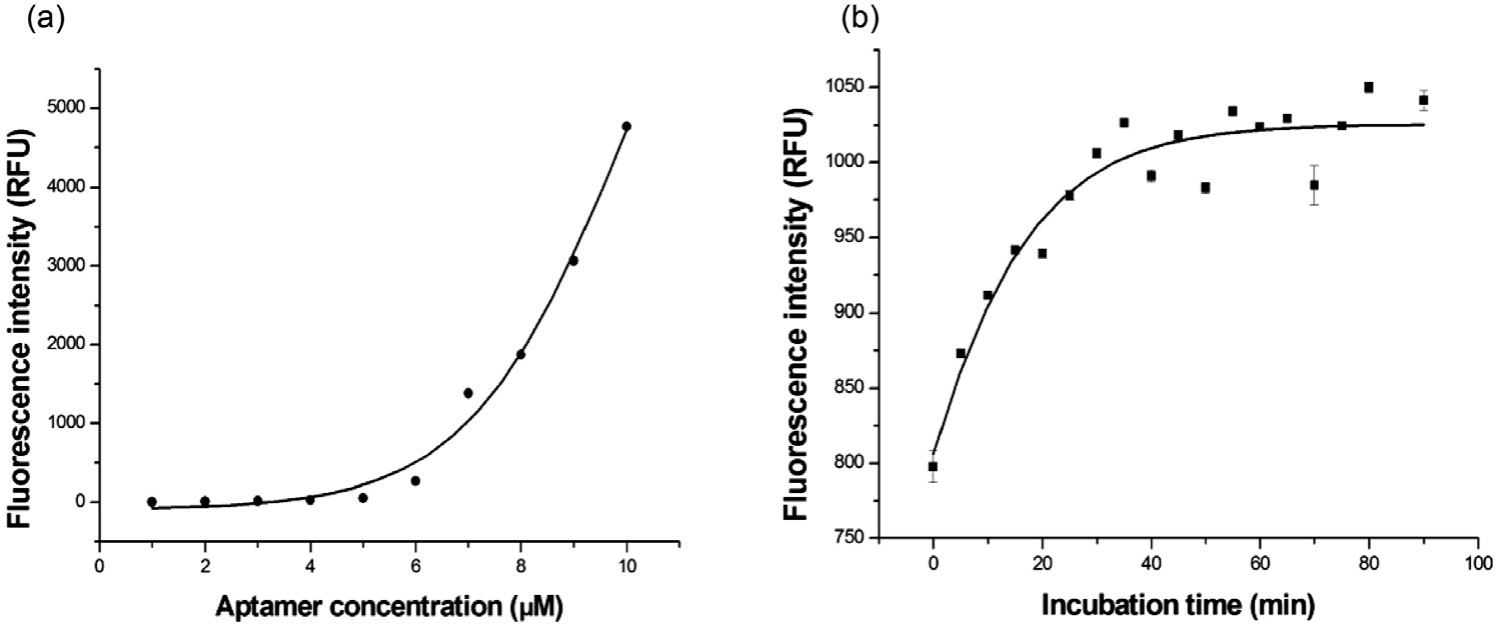
Fluorescence intensities of various concentrations of fluorescence-labeled H8 aptamer incubating with GO used the developed aptasensor (a) and (b) correlation of fluorescent intensities and incubation time of the developed platform.

The results of the optimization study show that 15 µL of 5 µM aptamer concentration completely quenches the fluorescence intensity (Fig. 1). Therefore, all experiments in this study are conducted using 5 µM aptamer concentration (stock solution). Based on the calculation of the maximum binding capacity of the aptamer, the result indicates that the aptasensor can analyze the HSA protein up to a maximum amount of 75 µmol (5 mg). Thus, it can be used for the detection of HSA in urine without sample dilution because the albuminuria concentration in normal urine is usually lower than 30 µg.mL^−1^ [3]. For the incubation time, the saturation of fluorescence intensity starts at 25 min and is kept constant until 90 min. Therefore, 30 min incubation times are used in all experiments. As the pH of human urine is 4–10, the pH values of the sensor system were measured after the addition of 2 µL of samples with pH of 4, 7, and 10 in the system and it was determined that the pH values of the present system were not significantly different. This indicates that the pH of 4–10 does not significantly affect the aptasensor system.

### Validation results

**Fig. 2:**
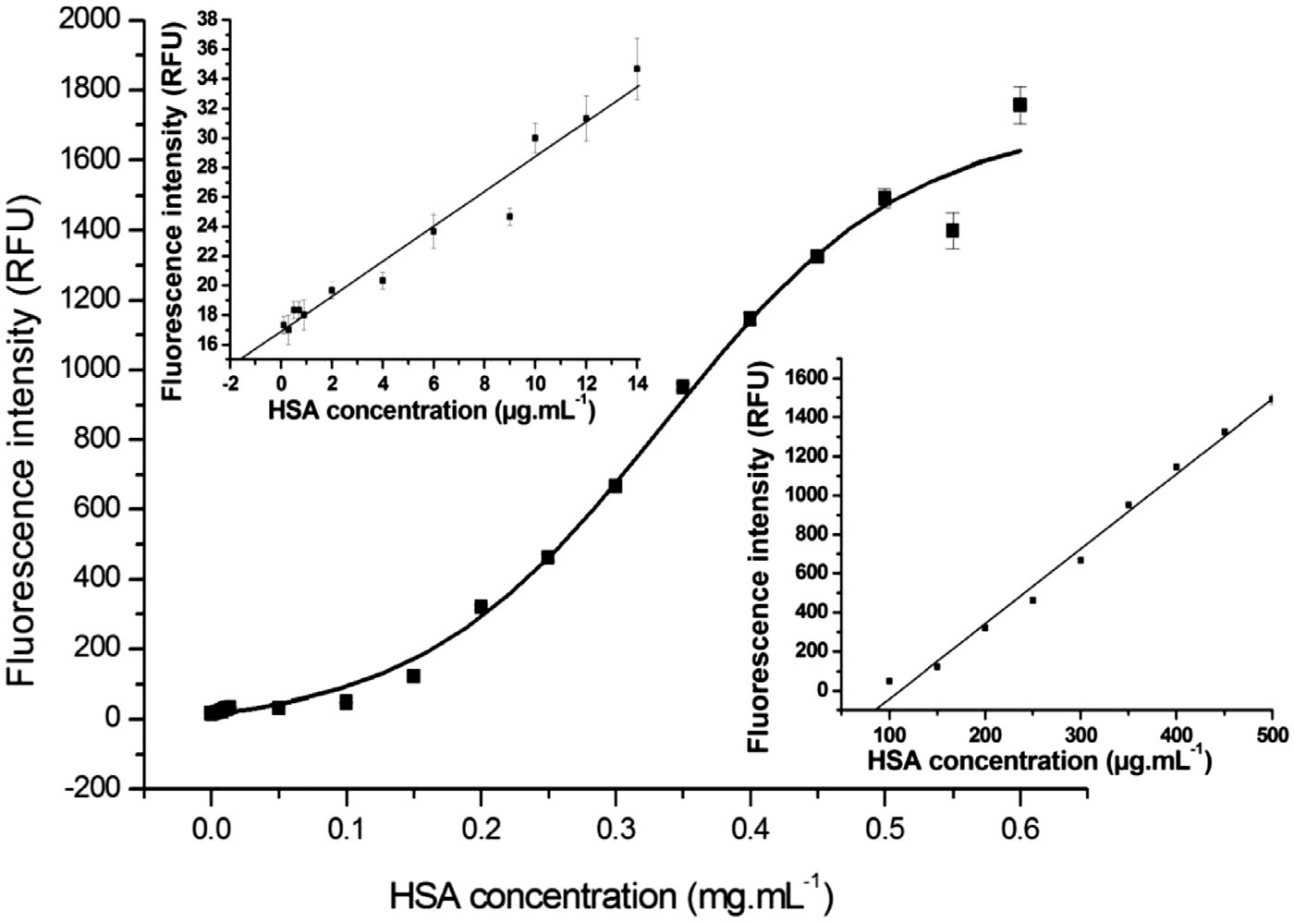
The correlation of fluorescence intensities and HSA concentrations in PBS buffer determined by the developed aptasensor. The middle graph is sigmoidal correlation of HSA concentration (0–0.6 mg.mL^−1^) and fluorescent intensity (R^2^ = 0.9814). The top graph is linear correlation of HSA concentration (0–14 µg.mL^−1^) and fluorescent intensity (R^2^ = 0.98918). The bottom graph is the linear correlation of HSA concentration (100–500 µg.mL^−1^) and fluorescent intensity (R^2^ = 0.99526).

Considering the sensitivity and specificity of the developed aptasensor, the calibration curve (Fig. 2) using the purified HSA protein in PBS buffer shows that the fluorescence intensity is sigmoidal correlated with HSA concentration at 0.1–600 µg.mL^−1^ (R^2^ = 0.989). In addition two linear standard curves were also observed, in which HSA concentrations were 0–14 µg.mL^−1^ (R^2^ = 0.98918) and 100–500 µg.mL^−1^ (R^2^ = 0.99526). The LOD of the developed platform was 0.05 µg.mL^−1^, which is 120-fold lower than the LOD of the standard immunoturbidimetry method (LOD of 6 µg.mL^−1^) [3]. In addition, the calibration curve using the purified HSA protein in artificial urine [4] shows the similar results as those in PBS buffer. These results indicate that this sensor has a high sensitivity and noble capability for HSA measurement in urine samples, which usually contain very low concentrations of HSA (<30 µg.mL^−1^) [5].

The developed aptasensor performance was compared with those of the standard immunoturbidimetry method used in hospitals by testing 120 urine samples (Table 1, Figure S1 and Table S1). The results show that albuminuria concentrations detected by the standard automated method are 3.0–221.3 µg.mL^−1^, whereas those detected by the developed aptasensor are 0.64–525.5 µg.mL^−1^. Considering the HSA concentrations of ≥0.1 mg.mL^−1^, the results show that the albuminuria concentrations determined by this developed aptasensor are significantly correlated with the data obtained by the standard automated method with *p* < 0.01 (r = 0.95). For the HSA concentrations of <0.1 mg.mL^−1^, the albuminuria concentrations obtained by the method proposed in this study are better differentiated than the values derived from the standard automated methods due to the lower LOD of the aptasensor [as described in the main paper].

**Table 1:** Performance of immunoturbidimetry automated method and graphene oxide-mediated fluorescence quenching aptasensor (120 urine samples).

Comparing items	Immunoturbidimetry	GO-mediated fluorescence quenching aptasensor
HSA Concentration	3.0–221.3 µg.mL^−1^	0.64–524.5 µg.mL^−1^
LOD	6 µg.mL^−1^	0.05 µg.mL^−1^
Operation time	5 min	30 min
Cost	$ 2	$ 0.3
Instrumentation	Automate	Automate and POCT

## Conclusion

In summary, a simple, cheap, and sensitive aptasensor is developed to quantify albuminuria in the range of 0.1–600 µg.mL^−1^, therefore no requirement of sample dilution. In addition, it can be applied for high-throughput albuminuria detection and as a portable POCT device. As the production cost is low, it has a significant potential for commercialization and public use.

## Appendix Supplementary materials

Image, application 1
