# Supplementary material for: Albuminuria detection using graphene oxide-mediated fluorescence quenching aptasensor
Source: MethodsX. 2020 Oct 20;7:101114. doi: 10.1016/j.mex.2020.101114 (PMC7644748; doi:10.1016/j.mex.2020.101114)
Supplement: Supplementary file 1 [file mmc1.pdf]

Submitted to MethodsX

Supporting Information

Albuminuria detection using graphene oxide-mediated fluorescence quenching aptasensor

Wireeya Chawjiraphan[a], Chayachon Apiwat[a,c], Khoonsake Segkhoonthod[a], Kiatnida Treerattrakoon[a,f], Preedee Pinpradup[a], Nuankanya Sathirapongsasuti[b], Prapasiri Pongprayoon[c,d], Patraporn Luksirikul[c.d], Patcharee Isarankura-Na-Ayudhya[e], Deanpen Japrung[a]*

[a]National Nanotechnology Center (NANOTEC), National Science and Technology Development Agency (NSTDA), Thailand Science Park, Pathumthani, Thailand

[b]Section for Translational Medicine, Faculty of Medicine Ramathibodi Hospital, Mahidol University, Bangkok, Thailand

[c]Department of Chemistry, Faculty of Science, Kasetsart University, Bangkok, Thailand

[d]Center for Advanced Studies in Nanotechnology for Chemical, Food and Agricultural Industries, KU Institute for Advanced Studies, Kasetsart University, Bangkok, Thailand.

[e]Department of Medical Technology, Faculty of Allied Health Science, Thammasat University, Pathumthani, Thailand

[f]Department of Pure and Applied Chemistry, Technology and Innovation Centre, University of Strathclyde, Glasgow, United Kingdom

*Corresponding author: deanpen@nanotec.or.th

Phone number: +66 2564 6985

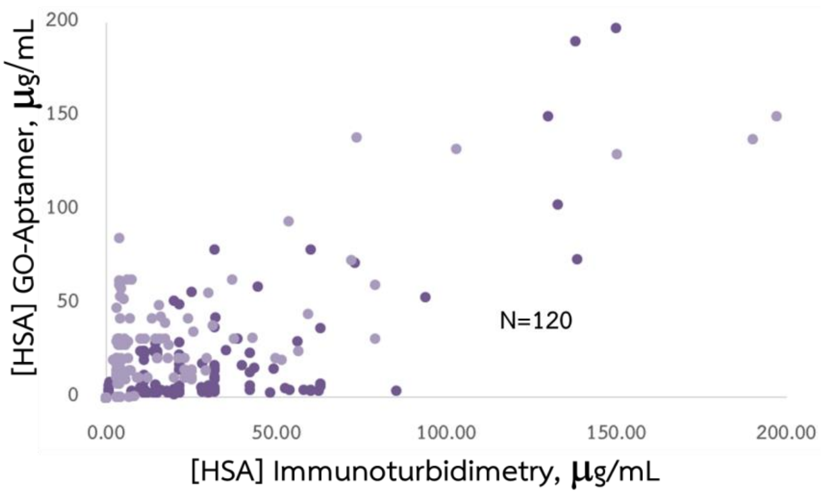

Figure S1: Correlation of the HSA concentrations (N=120) measured by graphene oxide-mediated fluorescence quenching aptasensor (y-axis) with those obtained by the standard methods, immunoturbidimetry (x-axis).

Table S1: Human Serum Albumin concentration (μg/mL) analyzed by our developed method comparing with immunotubidimetry method (Standard method used in the hospital)

**Urine sample number**	**Human serum albumin concentration (μg/mL)**		**Patient information**
	Our developed method	Immunotubidimetry method	
**1**	11.01	20	Healthy control (No DM)
**2**	1.00	8.3	Healthy control (No DM)
**3**	11.01	12.2	Healthy control (No DM)
**4**	0.64	6.6	Healthy control (No DM)
**5**	0.64	3	Healthy control (No DM)
**6**	10.00	5.7	Healthy control (No DM)
**7**	21.37	15	Healthy control (No DM)
**8**	21.37	18	Healthy control (No DM)
**9**	14.46	29.2	Healthy control (No DM)
**10**	31.74	10	Healthy control (No DM)
**11**	15.00	3	Healthy control (No DM)

**12**	56.22	30	Healthy control (No DM)
**13**	14.00	25	Healthy control (No DM)
**14**	15.00	23	Healthy control (No DM)
**15**	12.00	3	Healthy control (No DM)
**16**	31.74	17.2	Healthy control (No DM)
**17**	11.01	4.4	Healthy control (No DM)
**18**	42.11	13.4	Healthy control (No DM)
**19**	14.46	7.2	Healthy control (No DM)
**20**	28.00	5.6	Healthy control (No DM)
**21**	21.37	15	Healthy control (No DM)
**22**	14.46	6.7	Healthy control (No DM)
**23**	10.00	25	Healthy control (No DM)
**24**	11.01	25	Healthy control (No DM)
**25**	11.01	8.9	Healthy control (No DM)
**26**	21.37	21.3	Healthy control (No DM)
**27**	31.74	5.4	Healthy control (No DM)
**28**	49.22	15.4	Healthy control (No DM)
**29**	11.01	4.4	Healthy control (No DM)
**30**	20.00	3	Healthy control (No DM)
**31**	21.37	6.5	Healthy control (No DM)
**32**	11.01	4.7	Healthy control (No DM)
**33**	31.74	15.7	Healthy control (No DM)
**34**	28.29	18.4	Healthy control (No DM)
**35**	30.00	6.5	Healthy control (No DM)
**36**	20.00	4.6	Healthy control (No DM)

**37**	43.40	16	Healthy control (No DM)
**38**	21.37	3	Healthy control (No DM)
**39**	31.74	3.4	Healthy control (No DM)
**40**	31.74	3.8	Healthy control (No DM)
**41**	85.35	3.9	DM + Normal Kidney
**42**	0.64	3	DM + Normal Kidney
**43**	30.00	3	DM + Normal Kidney
**44**	31.74	37.6	DM + Normal Kidney
**45**	20.00	3	DM + Normal Kidney
**46**	28.29	4.4	DM + Normal Kidney
**47**	7.55	3.4	DM + Normal Kidney
**48**	58.16	4.2	DM + Normal Kidney
**49**	15.00	25	DM + Normal Kidney
**50**	42.11	6.8	DM + Normal Kidney
**51**	21.37	29.6	DM + Normal Kidney
**52**	11.01	4.9	DM + Normal Kidney
**53**	20.00	3	DM + Normal Kidney
**54**	31.74	14.5	DM + Normal Kidney
**55**	52.48	5.1	DM + Normal Kidney
**56**	11.01	25	DM + Normal Kidney
**57**	11.01	23.9	DM + Normal Kidney
**58**	11.01	3	Non DM+ Normal CKD
**59**	93.94	53.6	Non DM+ Normal CKD
**60**	31.74	79	Non DM+ Normal CKD
**61**	73.21	72	Non DM+ Normal CKD
**62**	138.56	73.7	Non DM+ Normal CKD
**63**	343.24	113.3	Non DM+ Normal CKD
**64**	20.00	51.6	Non DM+ Normal CKD
**65**	21.37	3	Non DM+ Normal CKD
**66**	60.00	79.1	Non DM+ Normal CKD

**67**	40.00	17.2	Non DM+ Normal CKD
**68**	44.56	59.3	Non DM+ Normal CKD
**69**	32.00	42.9	Non DM+ Normal CKD
**70**	62.84	7.5	Non DM+ Normal CKD
**71**	20.00	3	Non DM+ Normal CKD
**72**	11.01	3	Non DM+ Normal CKD
**73**	31.74	11.3	Non DM+ Normal CKD
**74**	42.11	24.1	Non DM+ Normal CKD
**75**	21.37	23.1	Non DM+ Normal CKD
**76**	38.65	31.3	Non DM+ Normal CKD
**77**	35.20	25.6	Non DM+ Normal CKD
**78**	62.84	6.1	Non DM+ Normal CKD
**79**	21.37	49.8	Non DM+ Normal CKD
**80**	48.06	3	Non DM+ Normal CKD
**81**	11.01	9.5	Non DM+ Normal CKD
**82**	11.01	22.9	Non DM+ Normal CKD
**83**	31.74	3.9	Non DM+ Normal CKD
**84**	42.11	4.1	Non DM+ Normal CKD
**85**	21.37	4.2	Non DM+ Normal CKD
**86**	60.10	4.1	Non DM+ Normal CKD
**87**	0.64	4.1	Non DM+ Normal CKD
**88**	28.29	3.5	Non DM+ Normal CKD
**89**	60.00	3.9	Non DM+ Normal CKD
**90**	53.89	4	Non DM+ Normal CKD
**91**	0.64	4	Non DM+ Normal CKD

**92**	0.64	3.6	Non DM+ Normal CKD
**93**	0.64	3.4	Non DM+ Normal CKD
**94**	11.01	3.9	Non DM+ Normal CKD
**95**	0.64	3.4	Non DM+ Normal CKD
**96**	0.64	3.8	Non DM+ Normal CKD
**97**	0.64	3.8	Non DM+ Normal CKD
**98**	20.00	4.3	Non DM+ Normal CKD
**99**	62.43	3.8	Non DM+ Normal CKD
**100**	17.92	3.8	Non DM+ Normal CKD
**101**	30.00	4.3	Non DM+ Normal CKD
**102**	7.55	3.9	Non DM+ Normal CKD
**103**	10.00	3.7	Non DM+ Normal CKD
**104**	62.04	221.3	DM with severe protein in urine
**105**	62.84	37	DM with severe protein in urine
**106**	100.00	204.5	DM with severe protein in urine
**107**	150.00	197.2	DM with severe protein in urine
**108**	138.00	190	DM with severe protein in urine
**109**	132.73	102.9	DM with severe protein in urine
**110**	130.00	150.1	DM with severe protein in urine
**111**	25.00	56.5	DM with severe protein in urine
**112**	19.00	3	Normoproteinuric DM + CKD
**113**	14.00	3	Normoproteinuric DM + CKD
**114**	16.00	4	Normoproteinuric DM + CKD
**115**	18.00	3	Normoproteinuric DM + CKD
**116**	20.00	2	Normoproteinuric DM + CKD

**117**	19.00	3	Normoproteinuric DM + CKD
**118**	524.47	36	Normoproteinuric DM + CKD
**119**	14.46	3.86	Normoproteinuric DM + CKD
**120**	12	3	Normoproteinuric DM + CKD
